# Effects of two different types of single exercise modes on salivary C-reactive protein concentration, oxidative stress and antioxidant capacity in post-myocardial infarction patients

**DOI:** 10.1080/13510002.2021.1890516

**Published:** 2021-02-22

**Authors:** Anna Gawron-Skarbek, Jacek Chrzczanowicz, Dariusz Nowak, Rafał Gawor, Tomasz Kostka

**Affiliations:** a Department of Hygiene and Health Promotion, Medical University of Lodz, Łódź, Poland; b Cardiac Rehabilitation Centre, Copernicus Memorial Hospital, Łódź, Poland; c Department of Clinical Physiology, Medical University of Lodz, Łódź, Poland; d Department of Geriatrics, Medical University of Lodz, Łódź, Poland

**Keywords:** Oxidative stress, inflammation, C-reactive protein, total antioxidant capacity, DPPH assay, saliva, exercise, cardiac rehabilitation

## Abstract

**Objectives:**

The aim of the study was to determine the effects of two different types of single cardiac rehabilitation (CR) exercise modes on the inflammation status, oxidative stress and total antioxidant capacity (TAC) of saliva.

**Methods:**

The study involved two groups of CR patients: group A (*n* = 21) used a cycloergometer, and group B (*n* = 21) received breathing and balance exercises. C-reactive protein as an inflammatory biomarker, malondialdehyde (MDA) as a measure of the level of oxidative stress and salivary 2.2-diphenyl-1-picryl-hydrazyl (DPPH) as an index of TAC were performed twice: before the beginning of the CR exercise (pre-CR) and immediately after (post-CR).

**Results:**

No significant changes were observed for the inflammatory response of saliva after CR exercise regardless of its type. MDA decreased (pre-CR: 39.7 ± 101.9 vs. post-CR: 16.8 ± 44.3 ng·mL^−1^; *p* < 0.01) and DPPH increased (pre-CR: 25.9 ± 16.7 vs. post-CR: 32.6 ± 14.0% reduction; *p* < 0.05) after CR exercise in the group B, with similar but not statistically significant changes in the group A.

**Discussion:**

Two popular exercise modes, especially breathing and balance exercises, reduce salivary oxidative stress and enhance the antioxidant potential of saliva in CR patients. The approval of saliva as a non-invasive source of information about inflammation status, oxidative stress and antioxidant capacity in cardiac patients requires further studies.

## 1. Introduction

Regular moderate physical exercises constitute a crucial element of cardiac rehabilitation (CR) in cardiovascular diseases patients. Although CR is known to have an overall beneficial effect [1,2], its impact on antioxidant status or oxidative stress is not well recognized.

Some data indicate that physical exercise may enhance antioxidant potential among patients with cardiovascular diseases [3,4], others present a lack of association [5,6]. It remains unresolved whether an increase in antioxidant properties of biological fluids (plasma, serum, saliva) results from an increased defence response against exercise-induced oxidative stress and the accompanying transient inflammatory reaction, or is rather a fixed adaptation response only in more physically active individuals [7].

CR plays a key role in the treatment of cardiac subjects by its anti-inflammatory effects; however, the effect of exercise on salivary concentration of C-reactive protein (CRP), as well as on the oxidative stress, expressed as malondialdehyde (MDA) concentration and total antioxidant capacity (TAC), has not been well studied [8–10]. Saliva is an easily obtainable body fluid for medical research and requires little effort for its collection. It has been proposed that saliva may offer potential as a biological fluid for biochemical monitoring of intra-body changes caused by physical exertion [11,12].

Cycloergometer training and exercises in the gym are the two popular modes of moderate exercise used in CR. Since various forms of physical activity may have different effects on serum antioxidant abilities or oxidative stress of cardiac patients, the type of cardiac rehabilitation may also have different effects on serum or salivary oxidant/antioxidant status.

Therefore, the aim of the study was to determine the effects of two widely-used different types of single acute CR exercise on the inflammation status, oxidative stress and total antioxidant capacity of saliva in post-myocardial infarction patients.

## 2. Materials and methods

### 2.1. Subjects

A total of 42 middle-aged and older (age range: 42.0–79.0 years) cardiovascular disease patients (74% male) participated in the study. The participants were randomly selected from a group of post-myocardial infarction patients qualified to the CR program in the Cardiac Rehabilitation Centre, Copernicus Memorial Hospital, Lodz (Poland). Twenty-one subjects (group A) were rehabilitated on a cycloergometer (age range: 42.0–71.0 years; 67% of male) and a further 21 (group B) not qualified for standard rehabilitation (age range: 47.0–79.0 years; 81% male) received breathing and balance exercises in the gym (multi-modal exercise program) The participants were assigned to group A or group B on the basis of physician’s order; taking into account the physical abilities and severity of patient’s disease.

Some individuals suffered from hypercholesterolemia (*n* = 20), arterial hypertension (*n* = 16), diabetes mellitus (*n* = 11), thyroid dysfunction (*n* = 2), chronic obstructive pulmonary disease (*n* = 1), and asthma (*n* = 1). All diagnosed disorders were in a stable phase and were pharmacologically controlled; pharmacological therapy in cardiovascular diseases usually included aspirin, beta-blockers, statins, and angiotensin-converting enzyme inhibitors.

Except for cholesterol, glucose and salt restrictions, none of the patients followed any special diet. Two of the male participants were smokers, one in either group; however, they reported smoking their last cigarette at least 12 h before the examination. The study was carried out following the rules of the Declaration of Helsinki, and the protocol was approved by the local ethics committee (RNN/73/15/KE).

### 2.2. Study design

The examinations were conducted in the Cardiac Rehabilitation Centre, Copernicus Memorial Hospital of Lodz. The laboratory measurements were performed in the Department of Clinical Physiology and in the Central Scientific Laboratory, both at the Medical University of Lodz. The patients reported to the centre for unstimulated saliva sample collection between 7.00 and 9.00 am after overnight fasting, not smoking, and resting for at least 12 h. They had previously been asked to clean their teeth after the last meal on the day preceding the examination.

Before the patients began the four-week CR program, each was tested for blood pressure, and heart rate at rest, and anthropometric, comorbidities, drug use, smoking and dietary habits were recorded. Height and weight were measured and the Body Mass Index (BMI) was calculated. Waist-to-Hip Ratio (WHR) was evaluated on the basis of waist and hip circumference. Salivary TAC, CRP, MDA parameters were assessed twice: once before the beginning of the first CR exercise (pre-CR exercise) and once immediately afterwards (post-CR exercise). Both types of CR exercises lasted up for 30 min; group A carried out CR interval exercise on a cycloergometer (Ergoline Reha 900, Bitz, Germany) with a load individually adapted to the patient and group B performed breathing and balance exercises in the gym.

### 2.3. Laboratory measurements

#### 2.3.1. Salivary pH

Saliva pH was determined immediately after sampling (±5 mL) using a standard colorimetric strip test. Following this, the saliva was centrifuged to separate all debris (10 min, 4°C, 1 125 g). The supernatant was stored at –80°C for a maximum of 30 days.

#### 2.3.2. Salivary CRP

Salivary CRP (ELISA Kit – Salimetrics, PA, USA) was determined based on the colorimetric CRP peroxidase reaction with tetramethylbenzidine as a substrate. Optical density was read on a standard VICTOR^TM^ X4 multifunctional microplate reader [Perkin Elmer] at a wavelength of 450 nm. The amount of CRP peroxidase detected was directly proportional to the CRP concentration (ng·mL^−1^) in the sample [13].

#### 2.3.3. Salivary MDA

A high-sensitivity ELISA kit (Cloud-Clone Corp, Katy, TX USA) was applied to evaluate the level of salivary MDA. The assay is based on a competitive inhibition reaction between biotin labelled MDA and unlabelled MDA (samples) with the pre-coated monoclonal antibody specific to MDA. The concentration of MDA (ng·mL^−1^) in the sample is inversely proportional to the amount of bound Horseradish Peroxidase conjugate added earlier. The final reading was at *λ* = 450 nm.

#### 2.3.4. Salivary TAC

Salivary TAC was measured spectrophotometrically using an Ultrospec III spectrophotometer with Spectro-Kinetics software (LKB Biochrom Pharmacia, Cambridge, England) by the 2.2-diphenyl-1-picryl-hydrazyl (DPPH) method [14]. This method is a promising approach for assessing antioxidant status in clinical studies; it measures the total activity of circulating low molecular-weight non-enzymatic antioxidants through their ability to decompose DPPH radicals. The results are expressed as a percentage of free-radical DPPH reduction. The details of the method are described elsewhere [15,16].

To enhance data reliability, all results were calculated as the mean of two separate experiments. The saliva values taken immediately following exercise (post-CR) were compared with those taken at the start of the exercise (pre-CR) to determine the change (i.e.: Δ CRP, Δ MDA, Δ DPPH, and Δ pH, respectively).

### 2.4. Statistical analysis

Data were verified for normality of distribution and equality of variance. Variables that did not meet the assumption of normality were analysed with non-parametric statistics. The one-way analysis of variance (ANOVA) and the Kruskal–Wallis test were used for comparison of pre-CR exercise and post-CR exercise values and changes (Δ) in CRP, MDA, DPPH, and pH values along with CR exercise between the groups A and B. The paired t-test and non-parametric sign test for pair values were used to compare the pre-CR and post-CR readings for all variables. The Pearson product moment and Spearman correlations were used to determine the relationships between numerical variables. The results for continuous variables were presented as mean ± SD. The level for statistical significance was set at *p* < 0.05. Statgraphics 5.0 (Statgraphics Technologies, Inc, the Plains, VA, USA) was used for statistical analysis.

## 3. Results

### 3.1. General characteristics

Group B demonstrated a higher mean age than group A (*p* < 0.001) and was characterized by higher HR values (*p* < 0.05). No other differences were identified between the two subgroups for the remaining parameters (Table 1). Over 1/5 of the study group were obese, almost a half were overweight, and more than half (64%) presented abdominal obesity. These results did not differ after excluding the smoker from each group.

**Table 1.** General characteristics of the whole study group and subgroups.

Variable	Study group (*n *= 42)	Group A (*n *= 21)	Group B (*n *= 21)
Age (years)	62.7 ± 10.2	57.1 ± 9.3	68.3 ± 7.8^‡^
BMI (kg·m^−2^)	27.7 ± 4.3	28.4 ± 5.0	27.0 ± 3.4
WHR	0.97 ± 0.09	0.96 ± 0.09	0.98 ± 0.07
SBP (mmHg)	127.6 ± 13.4	126.0 ± 10.6	129.3 ± 15.7
DBP (mmHg)	74.4 ± 11.1	77.0 ± 12.9	71.7 ± 8.5
HR (beats per minute)	65.3 ± 5.6	63.3 ± 4.9	67.2 ± 5.7*

BMI, body mass index; DBP, diastolic blood pressure, HR, heart rate; SBP, systolic blood pressure; WHR, waist-to-hip ratio.

^‡^ *p* ≤ 0.001.

*p < 0.05 (as compared to group A).

### 3.2. Comparison of pH, inflammatory, oxidant and antioxidant indexes of saliva between subgroups

Table 2 shows pre- and post-CR exercise values obtained for salivary CRP, MDA, DPPH and pH for the whole study group (*n* = 42) and according to type of CR exercise. Group A and group B presented similar pre- and post-CR exercise values for inflammatory and antioxidant indexes. Although no significant differences were found between the groups for the pre-CR MDA and pre-CR pH values, group B demonstrated significantly lower values of post-CR MDA (*p* < 0.01) and post-CR pH (*p* < 0.05) than group A.

**Table 2.** Comparison of salivary pH, CRP, oxidant and antioxidant values before and after CR exercise for the whole study group and two subgroups.

Variable	**Study group (*n*** **=** **42)**	**Group A (*n*** **=** **21)**	**Group B (*n*** **=** **21)**
CRP pre (ng·mL^−1^)	2.9 ± 4.0	1.8 ± 1.3	3.9 ± 5.4
CRP post (ng·mL^−1^)	2.3 ± 2.8	2.6 ± 3.6	1.8 ± 1.8
Δ CRP	−0.6 ± 4.1	0.8 ± 3.5	−2.1 ± 4.3
MDA pre (ng·mL^−1^)	39.5 ± 76.0	39.4 ± 38.3	39.7 ± 101.9
MDA post (ng·mL^−1^)	23.7 ± 44.7^b^	30.6 ± 45.2	16.8 ± 44.3^†,b^
Δ MDA	−15.8 ± 49.2	−8.8 ± 35.5	−22.8 ± 60.0
DPPH pre (% reduction)	26.4 ± 15.7	26.9 ± 14.9	25.9 ± 16.7
DPPH post (% reduction)	30.6 ± 14.0^a^	28.7 ± 14.0	32.6 ± 14.0^a^
Δ DPPH	4.2 ± 13.2	1.7 ± 12.0	6.6 ± 14.2
pH pre	6.8 ± 0.4	6.9 ± 0.4	6.7 ± 0.4
pH post	6.5 ± 0.5^b^	6.7 ± 0.5	6.3 ± 0.4^*,b^
Δ pH	−0.2 ± 0.3	−0.2 ± 0.4	−0.3 ± 0.3

CRP, C-reactive protein; DPPH, 2.2-diphenyl-1-picryl-hydrazyl; MDA, malondialdehyde; pre, examination before CR exercise; post, examination immediately after CR exercise.

^†^ *p* < 0.01, *p < 0.05 (as compared to group A); ^a^*p* < 0.05, ^b^*p* < 0.01 (as compared to pre-CR exercise values).

### 3.3. Post-CR exercise changes in salivary parameters

No change was observed between the pre- and post-CR exercise values for CRP in the whole study group (*n* = 42); however, post-CR MDA (*p* < 0.01) and post-CR pH (*p* < 0.01) were found to be significantly lower and post-CR DPPH (*p* < 0.05) significantly higher than their pre-CR exercise values (Table 2).

In group A, no significant change was observed for CRP, MDA, DPPH or pH (*p* > 0.05) after CR exercise.

In group B, similar to the whole group, DPPH (*p* < 0.05) significantly increased after training while MDA (*p* < 0.01) and pH (*p* < 0.01) fell; only CRP did not change.

### 3.4. Correlations with antioxidant and oxidant parameters of saliva (*n* = 42)

No correlations were found between anthropometric parameters and salivary antioxidant/oxidant indices, except for a single positive association between BMI and post-CR exercise MDA (rho = 0.40, *p* < 0.05). The overweight and obese patients demonstrated a higher level of oxidative stress after CR exercise.

Positive correlations were identified between pre-CR DPPH and post-CR DPPH (rho = 0.52, *p* < 0.001), pre- and post-CR CRP (rho = 0.73, *p* < 0.001), pre- and post-CR MDA (rho = 0.51, *p* < 0.001), and pre- and post-CR pH (r=0.64, *p* < 0.001).

### 3.5. Correlations with Δ DPPH, Δ CRP and Δ MDA (*n* = 42)

Lower pre-CR exercise DPPH values were associated with higher Δ DPPH (rho = −0.51, *p* < 0.01). This could be interpreted as the subjects with weaker basic antioxidant capacities display a stronger antioxidant reaction after CR exercise.

Conversely, higher pre-CR CRP was associated with lower Δ CRP values (rho = −0.48, *p* < 0.01). A greater drop of CRP after CR exercise was associated with a higher fasting pre-CR salivary CRP, i.e. with a higher tendency to inflammation of saliva. Similarly, subjects with a higher level of salivary oxidative stress at baseline demonstrated a greater decline of MDA (rho = −0.38, *p* < 0.05).

**Figure 1.**
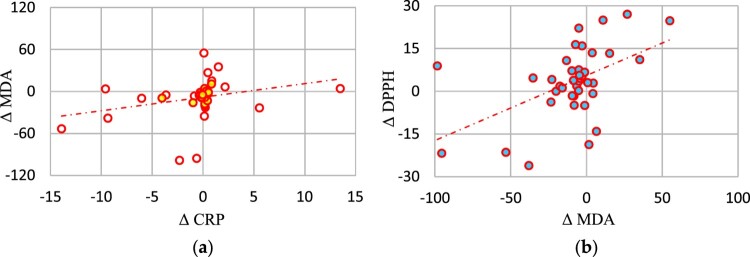
Correlations of Δ MDA with ΔCRP (a), and with Δ DPPH (b) in the study group (*n* = 42). CRP – C-reactive protein DPPH – 2.2-diphenyl-1-picryl-hydrazyl, MDA – malondialdehyde.

Higher Δ MDA was related to higher Δ CRP (rho = 0.40, *p* < 0.05) (Figure 1(a)) and higher Δ DPPH (rho = 0.32, *p* < 0.05) (Figure 1(b)).

## 4. Discussion

To the best of our knowledge our study is the first that simultaneously assesses salivary biomarkers of exercise-induced oxidative stress (MDA concentration), inflammation (CRP concentration) and TAC (% reduction of DPPH radical) in a group of post-myocardial infarction subjects. Our findings indicate that a single CR exercise/training session at the beginning of the CR training cycle caused an increase of salivary antioxidant potential assessed by the DPPH test and a decline of the level of oxidative stress in saliva as measured by MDA; however, no change in salivary inflammation evaluated by the CRP concentration (with a tendency to decrease) was revealed among post-myocardial infarction/CR patients. Moreover, a greater increase in oxidative stress (Δ MDA) was accompanied by a larger increase in inflammatory reaction level (Δ CRP) and a larger increase in antioxidant capacities of saliva environment (Δ DPPH).

CR exercise type affected the post-CR findings: the lower post-CR exercise MDA and higher post-CR DPPH were identified in patients rehabilitated in a form of breathing and balance mode of exercise; these changes were less noticeable in the group training on a cycloergometer. This indicates that for patients with initially weaker exercise capacities, even lighter training in the gym induces an intense antioxidant defence response, consequently reducing the level of oxidative stress in saliva, which was not the case in group exercising on the cycloergometer i.e. in the group with the assumption of better adaptability to physical effort at baseline. Maybe in a group more physically fit the antioxidant reaction was more balanced and less violent than in the second group, where the effect of a decrease in oxidative stress was more spectacular. Also only in the group rehabilitated in the gym the salivary pH significantly declined after a single CR exercise (i.e. saliva acidity has increased significantly). A greater decrease of salivary pH in group B also may indicate that the mechanisms responsible for maintaining homeostasis in saliva were less efficient in this group.

There is not much data on the effect of exercise/physical effort on antioxidant and oxidant level of salivary biomarkers, even less so in the group of cardiologic patients [17,18]. It has been proven that CR exercises are beneficial for post-myocardial infarction patients [19], but it remains unclear whether moderate CR exercise may enhance, or rather attenuate the physiological antioxidant potential. Saliva has been successfully used as a medium for the reflection of health status but the research in this area is still limited. Available data concerning the impact of physical exercises on the salivary antioxidant status and oxidative stress parameters in subjects with different health conditions are not unambiguous [20,21]. For instance, the intensity of the exercise bout (related to type of physical training) determines the value of TBARS (oxidative stress biomarker): the more intense exercise-bouts have significantly increased the salivary TBARS [22], while less intense exercises have not [23]. In addition, the variety of methods (their limitations; e.g. a lack of enzymatic antioxidants in acetonitrile deproteinized saliva sample during DPPH test preparation procedure [15]), parameters tested (individual antioxidants as enzymes, uric acid versus TAC) or time of sample collection (immediately after the end of exercise, or for example, 1 [24] or 3 h later [25]) make it difficult to drawn common conclusions. As an example in the study by Rodrigues de Araujo et al. [26] performed among soccer athletes subjected to an acute high-intensity interval exercise unclear effects for i.a. MDA, catalase, and superoxide dismutase (SOD) were observed while uric acid (a main TAC contributor) showed a large decrease after physical effort (contrary to our results). Meanwhile, the data of the study by Coelho-Júnior et al. [27] indicate that an acute session of power and resistance exercise can be effective to cause beneficial changes on hemodynamic parameters and nitric oxide levels in older women, similarly as in salivary samples from young male subjects performing various type exercises (high-intensity interval, continuous, and resistance exercises) [25]. In the study by Souza et al. [25], healthy men demonstrated an increase of TAC measured by ferric-reducing antioxidant power (FRAP) analysis at the end of training in all different exercise protocols. In turn, in a group of young non-smoking women DPPH activity was significantly decreased immediately after exhaustive aerobic exercise [28]. As discussed by Munther [29] physical exercise may alter total salivary antioxidants activity; a higher total salivary antioxidants concentration was found among those who exercised compared to those who did not. Mendoza-Núñez et al. [30] reported that Tai Chi exercise during a period of 6 months increased SOD activity and total antioxidant status in saliva, as well as decreased the level of IL-1β (inflammation biomarker) in older adults. Similar effect was observed in patients with coronary artery disease after 6–8 weeks of moderate intensity aerobic exercise training: salivary high-sensitivity CRP level decreased after 24 sessions of CR [31] but these are examples of distant effects. In the present study after only a single CR exercise CRP concentration revealed a tendency to decrease.

Study limitations of the present study should be pointed. Pharmacotherapy and its heterogeneity could interfere with the results. It was not feasible to find CR patients free from common age-related ailments or using similar drugs and treatment regimens. Lack of detailed energy expenditure calculation for each patient can be seen as a restriction; however, it is not necessary to clearly differentiate between group A and B. A future examination could also include an assessment of oral health to evaluate a possible inflammatory effect in active caries or periodontitis cardiac patients on antioxidant/oxidant parameters. In addition, the study is based on a relatively limited number of subjects. The aim of our study was not the assessment of diagnostic reliability of the measurement of oxidative stress markers in saliva; however, a several previous papers have presented the data of utility of saliva for assessing these biochemical indices [14,18,23,32]. Nevertheless further clinical studies should involve sex- and age-matched groups, with a larger number of subjects to verify the exercise-induced possible antioxidant/oxidant changes in saliva and confirm the present findings with serum results.

## 5. Conclusions

A single CR exercise reduces salivary oxidative stress and enhances antioxidant potential of saliva in post-myocardial infarction/CR patients receiving a breathing and balance exercise modes; however, the inflammatory response is more or less unchanged regardless of CR exercise type. A greater intensification of oxidative stress in saliva, generated as a result of exercise overload during a single CR training unit, might be muted by the simultaneous strengthening of the salivary antioxidant capacity. The approval of saliva as a non-invasive source of information about inflammation status, oxidative stress and antioxidant capacity in cardiac patients requires a simultaneous assessment and confirmation of serum results of CRP, oxidative status and TAC with the findings of salivary parameters.

## References

[1] Kato M, Kubo A, Nihei F, et al. Effects of exercise training on exercise capacity, cardiac function, BMI, and quality of life in patients with atrial fibrillation: a meta-analysis of randomized-controlled trials. Int J Rehabil Res. 2017;40:193–201. doi:10.1097/mrr.0000000000000232. 28796004

[2] Kubilius R, Jasiukeviciene L, Grizas V, et al. The impact of complex cardiac rehabilitation on manifestation of risk factors in patients with coronary heart disease. Medicina (Kaunas). 2012;48:166–173. 22588350

[3] Garza MA, Wason EA, Zhang JQ. Cardiac remodeling and physical training post myocardial infarction. World J Cardiol. 2015;7:52–64. doi:10.4330/wjc.v7.i2.52. 25717353 PMC4325302

[4] Chrzczanowicz J, Gawron-Skarbek A, Kostka J, et al. Physical activity and total antioxidant capacity across an adult lifespan of men. Med Sci Sports Exerc. 2012;44:575–582. doi:10.1249/MSS.0b013e318238b7f0. 21952634

[5] Demirbag R, Yilmaz R, Kocyigit A. Relationship between DNA damage, total antioxidant capacity and coronary artery disease. Mutat Res. 2005;570:197–203. doi:10.1016/j.mrfmmm.2004.11.003. 15708578

[6] Smith DT, Carr LJ, Dorozynski C, et al. Internet-delivered lifestyle physical activity intervention: limited inflammation and antioxidant capacity efficacy in overweight adults. J Appl Physiol (1985). 2009;106:49–56. doi:10.1152/japplphysiol.90557.2008. 19008491 PMC2636943

[7] El Abed K, Rebai H, Bloomer RJ, et al. Antioxidant status and oxidative stress at rest and in response to acute exercise in judokas and sedentary men. J Strength Cond Res. 2011;25:2400–2409. doi:10.1519/JSC.0b013e3181fc5c35. 21869626

[8] Al-Rawi NH, Shahid AM. Oxidative stress, antioxidants, and lipid profile in the serum and saliva of individuals with coronary heart disease: is there a link with periodontal health? Minerva Stomatol. 2017;66:212–225. doi:10.23736/s0026-4970.17.04062-6. 28707864

[9] Ho E, Karimi Galougahi K, Liu CC, et al. Biological markers of oxidative stress: applications to cardiovascular research and practice. Redox Biol. 2013;1:483–491. doi:10.1016/j.redox.2013.07.006. 24251116 PMC3830063

[10] Hendrix J, Nijs J, Ickmans K, et al. The interplay between oxidative stress, exercise, and pain in health and disease: potential role of autonomic regulation and epigenetic mechanisms. Antioxidants (Basel). 2020;9. doi:10.3390/antiox9111166. PMC7700330 33238564

[11] Papacosta E, Nassis GP. Saliva as a tool for monitoring steroid, peptide and immune markers in sport and exercise science. J Sci Med Sport. 2011;14:424–434. doi:10.1016/j.jsams.2011.03.004. 21474377

[12] Nam Y, Kim YY, Chang JY, et al. Salivary biomarkers of inflammation and oxidative stress in healthy adults. Arch Oral Biol. 2019;97:215–222. doi:10.1016/j.archoralbio.2018.10.026. 30399508

[13] Chard T. An introduction to radioimmunoassay and related techniques. Amsterdam: Elsevier Science; 1995; Vol. 6.

[14] Gawron-Skarbek A, Prymont-Przymińska A, Sobczak A, et al. A comparison of native and non-urate total antioxidant capacity of fasting plasma and saliva among middle-aged and older subjects. Redox Rep. 2018;23:57–62. doi:10.1080/13510002.2017.1392714. 29088986 PMC6748680

[15] Chrzczanowicz J, Gawron A, Zwolinska A, et al. Simple method for determining human serum 2,2-diphenyl-1-picryl-hydrazyl (DPPH) radical scavenging activity - possible application in clinical studies on dietary antioxidants. Clin Chem Lab Med. 2008;46:342–349. doi:10.1515/cclm.2008.062. 18254708

[16] Gawron-Skarbek A, Chrzczanowicz J, Kostka J, et al. Cardiovascular risk factors and total serum antioxidant capacity in healthy men and in men with coronary heart disease. Biomed Res Int. 2014;2014:216964. doi:10.1155/2014/216964. 25180177 PMC4144148

[17] Nguyen TT, Ngo LQ, Promsudthi A, et al. Salivary lipid peroxidation in patients with generalized chronic periodontitis and acute coronary syndrome. J Periodontol. 2016;87:134–141. doi:10.1902/jop.2015.150353. 26313018

[18] Smriti K, Pai KM, Ravindranath V, et al. Role of salivary malondialdehyde in assessment of oxidative stress among diabetics. J Oral Biol Craniofac Res. 2016;6:41–44. doi:10.1016/j.jobcr.2015.12.004. 26937368 PMC4756067

[19] Lavie CJ, Milani RV. Cardiac rehabilitation and exercise training in secondary coronary heart disease prevention. Prog Cardiovasc Dis. 2011;53:397–403. doi:10.1016/j.pcad.2011.02.008. 21545925

[20] Evans LW, Omaye ST. Use of saliva biomarkers to monitor efficacy of vitamin C in exercise-induced oxidative stress. Antioxidants (Basel). 2017;6. doi:10.3390/antiox6010005. PMC5384169 28085082

[21] Babaei P, Damirchi A, Soltani Tehrani B, et al. Effect of exercise training on saliva brain derived neurotrophic factor, catalase and vitamin c. Med J Islam Repub Iran. 2016;30:452. 28491827 PMC5419233

[22] Sant’anna M, Casimiro-Lopes G, Boaventura G, et al. Anaerobic exercise affects the saliva antioxidant/oxidant balance in high-performance pentathlon athletes. Human Mov. 2016;17:50–55.

[23] Deminice R, Sicchieri T, Payão PO, et al. Blood and salivary oxidative stress biomarkers following an acute session of resistance exercise in humans. Int J Sports Med. 2010;31:599–603. doi:10.1055/s-0030-1255107. 20617486

[24] Atsumi T, Iwakura I, Kashiwagi Y, et al. Free radical scavenging activity in the nonenzymatic fraction of human saliva: a simple DPPH assay showing the effect of physical exercise. Antioxid Redox Signal. 1999;1:537–546. doi:10.1089/ars.1999.1.4-537. 11233150

[25] Souza AV, Giolo JS, Teixeira RR, et al. Salivary and plasmatic antioxidant profile following continuous, resistance, and high-intensity interval exercise: preliminary study. Oxid Med Cell Longev. 2019;2019:5425021. doi:10.1155/2019/5425021. 31885802 PMC6899261

[26] Rodrigues de Araujo V, Lisboa P, Boaventura G, et al. Acute high-intensity exercise test in soccer athletes affects salivary biochemical markers. Free Radic Res. 2018;52:850–855. doi:10.1080/10715762.2018.1481288. 30027785

[27] Coelho-Junior HJ, Irigoyen MC, Aguiar SDS, et al. Acute effects of power and resistance exercises on hemodynamic measurements of older women. Clin Interv Aging. 2017;12:1103–1114. doi:10.2147/cia.s133838. 28744114 PMC5513809

[28] Arazi H, Taati B, Rafati Sajedi F, et al. Salivary antioxidants status following progressive aerobic exercise: what are the differences between waterpipe smokers and non-smokers? Antioxidants (Basel). 2019;8. doi:10.3390/antiox8100418. PMC6826468 31546982

[29] Munther S. The effects of cigarette smoking and exercise on total salivary antioxidant activity. Saudi Dent J. 2019;31:31–38. doi:10.1016/j.sdentj.2018.09.002. 30705566 PMC6349960

[30] Mendoza-Núñez VM, Hernández-Monjaraz B, Santiago-Osorio E, et al. Tai Chi exercise increases SOD activity and total antioxidant status in saliva and is linked to an improvement of periodontal disease in the elderly. Oxid Med Cell Longev. 2014;2014:603853. doi:10.1155/2014/603853. 24790703 PMC3984794

[31] Jamshidpour B, Moghadam BA, Vasaghi-Gharamaleki B, et al. The effects of phase III cardiac rehabilitation in serum and salivary Hs-CRP and anthropometric measurements in patients with coronary artery disease. J Contemp Dent Pract. 2013;14:819–824. doi:10.5005/jp-journals-10024-1409. 24685781

[32] Gawron-Skarbek A, Kontarska-Krauza M, Dynowska B, et al. Salivary and plasma native and non-urate total antioxidant capacity versus oral health status in older non-smoking adults. Arch Oral Biol. 2019;107:104515. doi:10.1016/j.archoralbio.2019.104515. 31442934

